# Excitability, synaptic balance, and addiction: The homeostatic dynamics of ionotropic glutamatergic receptors in VTA after cocaine exposure

**DOI:** 10.1186/s12993-020-00168-4

**Published:** 2020-06-11

**Authors:** Thiago C. Moulin, Helgi B. Schiöth

**Affiliations:** 1 Functional Pharmacology Unit, Department of Neuroscience, Uppsala University, Uppsala, Sweden; 2 Institute for Translational Medicine and Biotechnology, Sechenov First Moscow State Medical University, Moscow, Russia

**Keywords:** Drug dependence, Withdrawal, Homeostatic plasticity, Synaptic scaling, Dopamine

## Abstract

Glutamatergic AMPA and NMDA receptors in the ventral tegmental area (VTA) are central for cocaine first exposure and posterior craving maintenance. However, the exact rules that coordinate the synaptic dynamics of these receptors in dopaminergic VTA neurons and behavioral outcomes are poorly understood. Additionally, synaptic homeostatic plasticity is present in response to chronic excitability changes in neuronal circuits, adjusting the strength of synapses to stabilize the firing rate. Despite having correspondent mechanisms, little is known about the relationship between continuous cocaine exposure and homeostatic synaptic changes in the VTA neurons. Here, we assess the role of homeostatic mechanisms in the neurobiology of cocaine addiction by providing a brief overview of the parallels between cocaine-induced synaptic potentiation and long-term synaptic adaptations, focusing on the regulation of GluA1- and GluN1- containing receptors.

## Introduction

Dopaminergic projections originated in the ventral tegmental area (VTA) are critical for reward learning and, consequently, drug abuse behaviors [5, 19, 23]. Animal models of cocaine addiction are characterized by compulsive drug-seeking and drug-taking even after prolonged periods of withdrawal [4, 11]. A central hypothesis is that these craving phenotypes reflect greater incentive motivation for the drug and associated stimuli [21] mediated by the potentiation of glutamatergic synapses on VTA dopamine neurons [13]. For example, VTA dopamine neurons exhibit transient NMDA receptor (NMDAR)-dependent increases in AMPA receptor (AMPAR)-mediated currents following either single or repeated cocaine injections [2, 26]. Similarly, cocaine has been shown to facilitate the experimental induction of long-term potentiation (LTP) and enhance the responsiveness of dopamine neurons to AMPA administration [29]. Despite the broad knowledge on these mechanisms, the scientific literature does not provide details on how this synaptic reinforcement can lead to excitatory over-potentiation of the reward circuitry, which in turn may trigger synaptic homeostatic mechanisms [24].

The homeostatic regulation of synapses is a extensively studied process [17], believed as necessary for the adequate development and function of neuronal networks [25]. It is defined as a negative feedback response mechanism to chronically elevated or reduced activity in a neural circuit, where individual neurons adapt to these changes by modifying their synaptic excitability threshold. It can be achieved through adjustments in ionotropic glutamatergic receptors synaptic modulation [7, 18]. During homeostatic regulation, AMPAR and NMDAR numbers at the postsynaptic surface are scaled down- or upwardly in response to activity overexcitation or inhibition, respectively, presumably via changing trafficking processes, including insertion and internalization of the receptors [27].

Homeostatic mechanisms have been shown to regulate many aspects of brain function by the interaction with other types of plasticity [1, 16], and to influence the pathophysiology of neuropsychiatric and neurologic disorders [9]. However, the role of synaptic homeostasis in substance abuse and addiction was not yet investigated. This article considers recent evidence on the influence of synaptic potentiation and subsequent homeostatic regulation in the VTA into behavioral outcomes of cocaine craving after chronic drug exposure. We use the regulation of GluA1- and GluN1-containing glutamatergic channels as a framework to examine the interactions of these two types of synaptic plasticity and their influence on craving behavior.

## GluA1/GluN1 dynamics during cocaine exposure, re-exposure, and withdrawal

NMDARs and AMPARs are necessary for the acute cocaine-induced synaptic potentiation in VTA DA cells. By preventing the synthesis of the AMPAR subunits GluA1 or GluA2 [8], or the NMDAR subunit GluN1 [30] in cells expressing the DA transporter, cocaine-induced reinforcement can be prevented in glutamatergic synapses of the VTA DA cells. Interestingly, however, at the behavioral level these mutations are unable to change the locomotor sensitization following repeated cocaine exposure in mice.

Additionally, GluA1-containing AMPARs were shown to have a singular pattern of expression after cocaine exposure: they are up-regulated after acute, but not chronic, cocaine self-administration; however, the withdrawal period is able to induce GluA1-AMPARs up-regulation again [3, 14, 15]. Moreover, the expression of this subunit is related to cocaine intake motivation, as rats transiently over-expressing GluA1-AMPARs were shown to have increased craving in the progressive ratio habituation. In this paradigm, animals are trained to self-administer cocaine, but the number of necessary lever presses exponentially increases with each cocaine release. When analyzing the breaking point parameter, defined by the highest ratio of responses per injection achieved before a 1 h-period when no further injections were earned, rats overexpressing GluA1-AMPARs much took longer to reach this point when compared to controls [3].

This state-dependent expression pattern of GluA1-AMPARs was shown to be regulated by GluN1-containing NMDARs. By using a viral-mediated expression of a dominant-negative GluN1 subunit in VTA, it was demonstrated that the absence of this NMDA unit blocks the increase in GluA1-AMPARs after chronic cocaine intake [12]. Nevertheless, the behavioral effects of inhibiting GluN1 expression are somewhat unexpected. GluN1-negative animals have attenuated locomotor sensitization after withdrawal [30] and are unable to the reinstatement of cocaine-conditioned place preference [8]; however, transient absence of GluN1 applied to the VTA during chronic cocaine self-administration paradoxically enhances cocaine-seeking behavior [12]. After withdrawal, these rats take longer to reach the breaking point and earn more cocaine injections when compared to controls. Extinction of this behavior was also impaired, and the reinstatement of the drug-paired lever response was facilitated.

These counterintuitive results could only be explained after studying the influence of homeostatic regulations due to the continuous change in neuronal excitability induced by the transient manipulation of GluN1-NMDARs in VTA. In fact, it was demonstrated that transient GluN1 inactivation for 3weeks enhanced AMPAR-mediated locomotor activity and membrane expression of glutamate receptor subunits, including GluA1. The overexpression of these AMPA channels, in turn, drove an increased motivation for cocaine in a progressive ratio testing and kept elevated the cocaine-seeking behavior elicited in both extinction and cocaine-primed reinstatement, even after 3–5 weeks of withdrawal [12]. This body of evidence suggests that the homeostatic regulation of AMPAR is able to modulate cocaine craving during chronic cocaine exposure and withdrawal.

## The role of homeostatic plasticity

These results were interpreted as a specific explanation for the observed cocaine-seeking after transient GluN1-NMDARs inactivation. However, we believe these results also indicate that cocaine-induced plasticity may induce synaptic scaling processes in the VTA at more general conditions. For example, homeostatic plasticity mechanisms have been shown to modulate the pathophysiology of some neurologic and neuropsychiatric disorders, such as intellectual disability [22], Rett syndrome [20], schizophrenia [6], and Alzheimer’s disease [28].

Moreover, when investigating the role of VTA in a social defeat stress model of depression, it was observed that VTA dopaminergic neurons of depression-resilient mice display enhanced inhibitory currents as a homeostatic response to this behavioral challenge [10]. The study also demonstrated that by artificially activating of VTA DA neurons in depression-susceptible animals, self-tuning homeostatic compensations could be triggered, changing the behavior. Thus, by naturally or experimentally establishing homeostatic balance in VTA dopaminergic neurons, they become more stable in response to environmental perturbations.

**Fig. 1:**
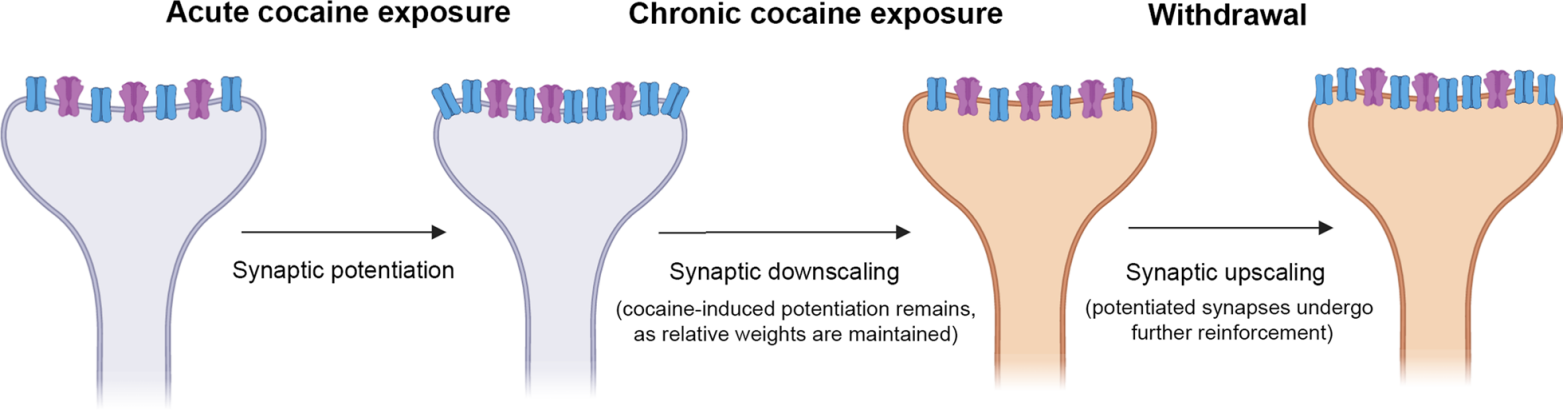
Schematics of the homeostatic-like characteristics of AMPAR-GluA1 dynamics after cocaine self-administration

When we look at cocaine-induced GluA1 plasticity in VTA neurons, after organizing by periods of drug intake and withdrawal (Fig. 1), we can observe a stereotypical process of AMPAR homeostatic regulation driven by enhanced excitation (chronic cocaine administration), followed by adaptation to decreased excitation (withdrawal). The acute intake of cocaine leads to short-term synaptic potentiation and enhanced expression of GluA1 proteins. However, after chronic intake of cocaine, GluA1 expression is comparable to that of controls, indicating a homeostatic response to continuous self-administration of the drug. Furthermore, during cocaine withdrawal, when the overall excitability is decreased, GluA1 levels rise again. These compensatory dynamics of AMPAR are consistent with homeostatic synaptic processes [24].

## Conclusions

To the best of our knowledge, this is the first report suggesting a connection between synaptic scaling processes and the physiological mechanisms of cocaine addiction in the VTA. Unquestionably, homeostatic regulations of VTA neurons are not the only responsible for craving behavior, as classic potentiation mechanisms and context reinforcement are also part of this process. In fact, most of the aforementioned cocaine-induced AMPAR/NMDAR plasticity takes place in VTA after cocaine self-administration, but not with passive yoked infusion, indicating a protagonist of learning and memory mechanisms. However, these recent results show that the contributions of synaptic homeostatic scaling in cocaine addiction must be further investigated. We believe such discussion would advance the understanding of homeostatic plasticity in behaviorally relevant in vivo models of addiction and provide further insight into the physiological underpinnings of compulsion and craving.

## Data Availability

Data sharing is not applicable to this article as no datasets were generated or analyzed during the current study.
